# Supplementary figures and images for: Dynamic recycling of extracellular ATP in human epithelial intestinal cells
Source: PLoS Comput Biol. 2023 Jun 29;19(6):e1011196. doi: 10.1371/journal.pcbi.1011196 (PMC10337955; doi:10.1371/journal.pcbi.1011196)

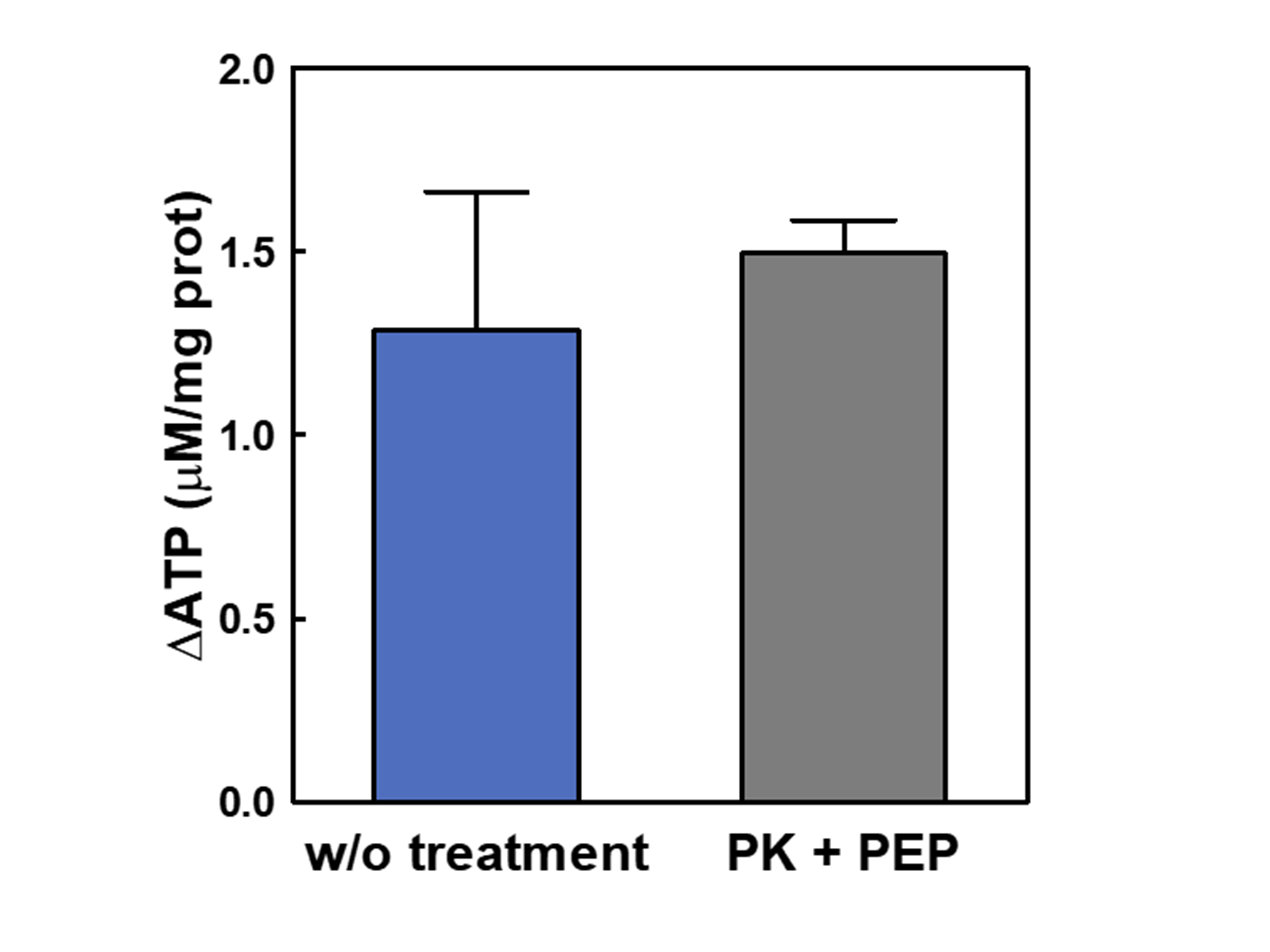

Supplement: S1 Fig — Increase in [eATP] after a 180 mOsm hypotonic shock in absence (blue) or presence (grey) of PK (3 U) and PEP (100 μM) were evaluated as ΔATP, i.e., the difference between [eATP] at 1 min post-stimulus and basal [eATP]. (TIF) [file pcbi.1011196.s001.tif]

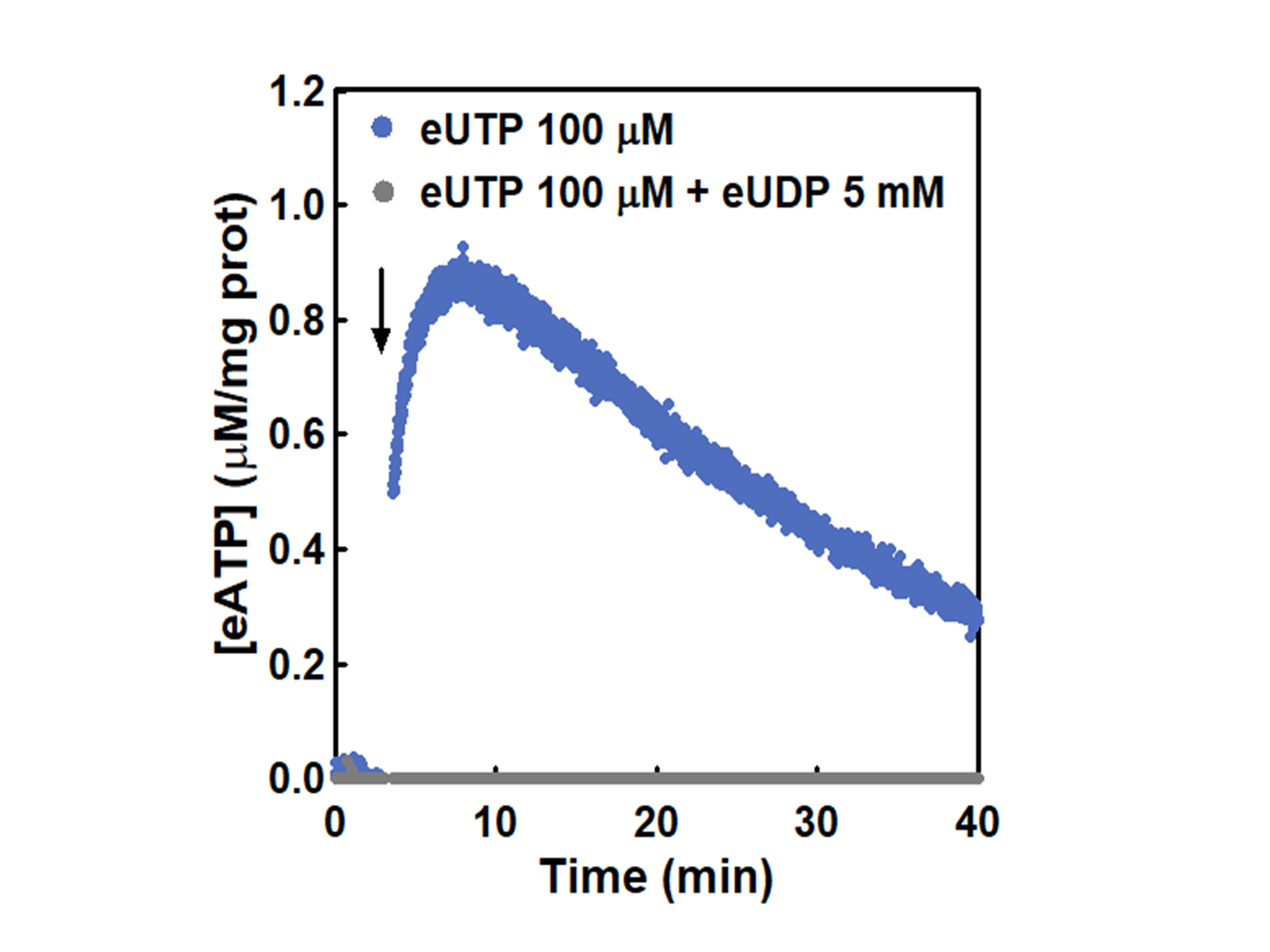

Supplement: S2 Fig — Time course of eATP accumulation in the presence of 100 eUTP μM in the absence (blue) or in the presence of 5 mM eUDP (grey). The data showed are the means of 3 independent experiments. (TIF) [file pcbi.1011196.s002.tif]

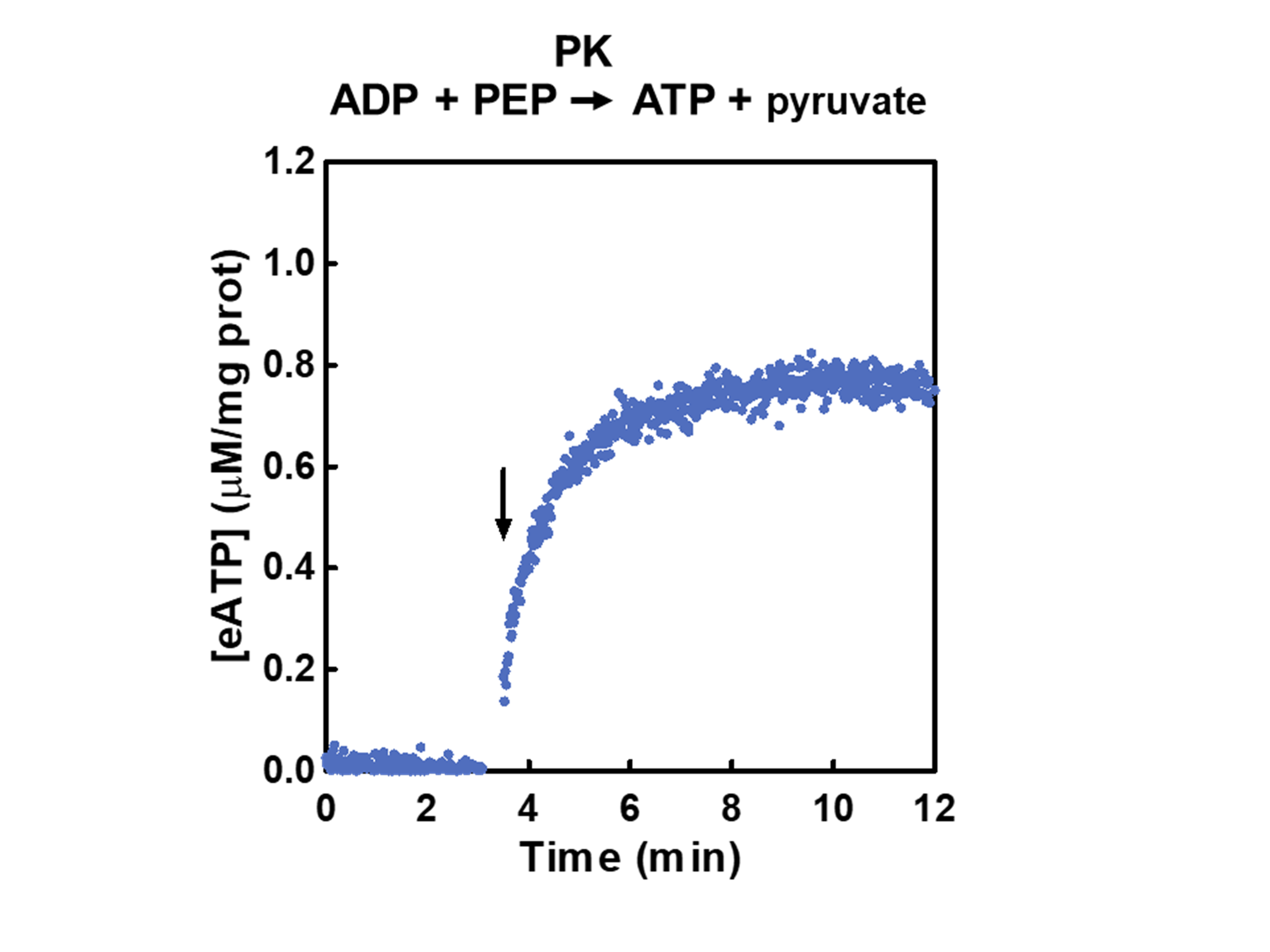

Supplement: S3 Fig — Caco-2 cells were incubated with luciferin-luciferase and, at the time indicated with the arrow, PK (3 U) and PEP (100 μM) were added. The value of the [eADP] in resting conditions was 0.77 ± 0.44 μM eADP/mg. Given a usual protein cell mass of 0.2 mg, the [eADP] in resting conditions is 0.15 ± 0.09 μM. The data showed are the means of 5 independent experiments. (TIF) [file pcbi.1011196.s003.tif]

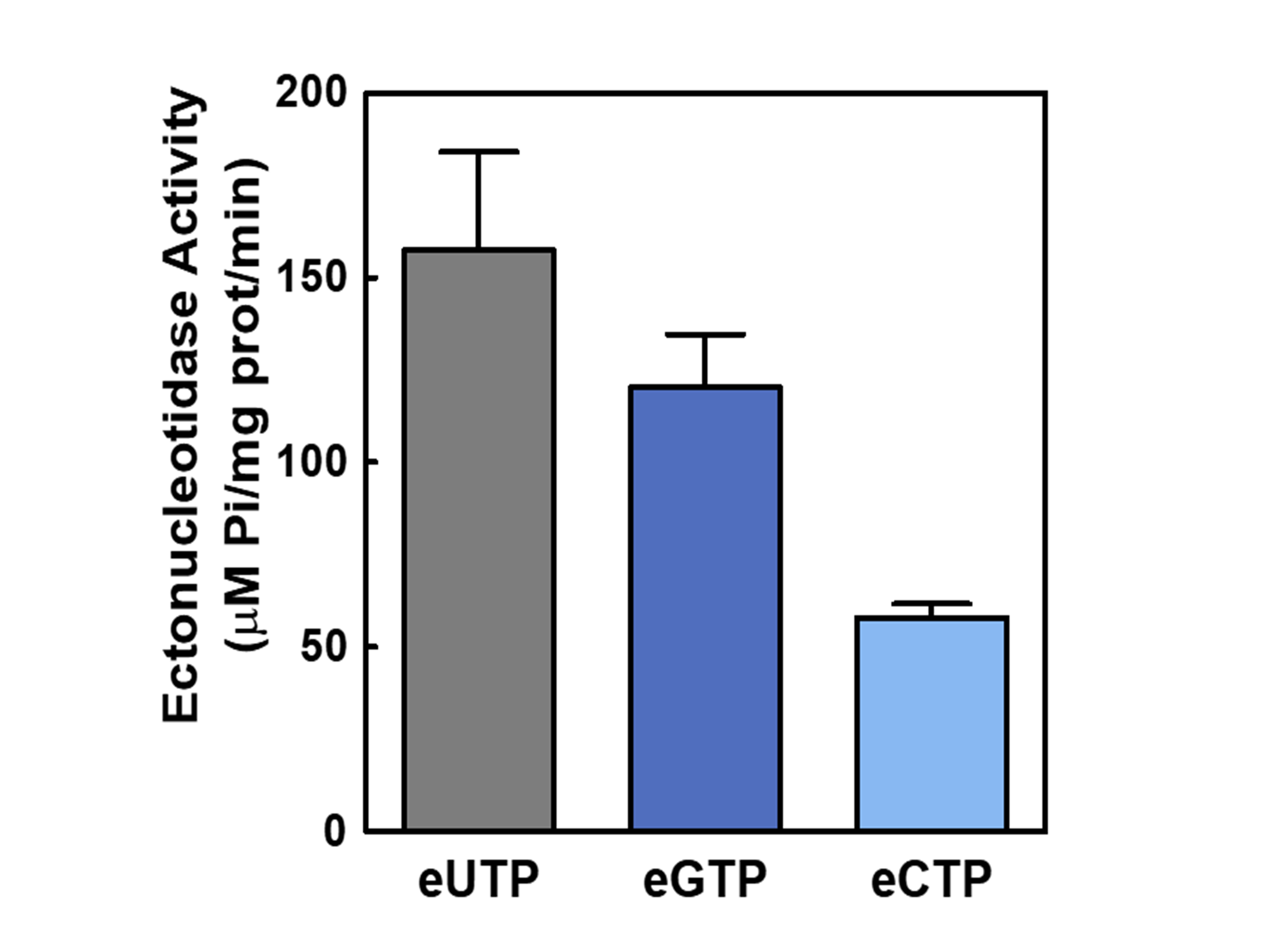

Supplement: S4 Fig — Experiments were performed in assay medium without Pi at room temperature, and Pi production was measured by the malachite green method (section 4.9.4). The time course of Pi accumulation in the extracellular media of Caco-2 cells was measured and values of enzyme activity were derived from initial rates of nucleotides hydrolysis for 500 μM of eUTP (grey), eGTP (blue) and eCTP (light blue). The data are the means of ± s.e.m. from 3 to 5 independent experiments. (TIF) [file pcbi.1011196.s004.tif]

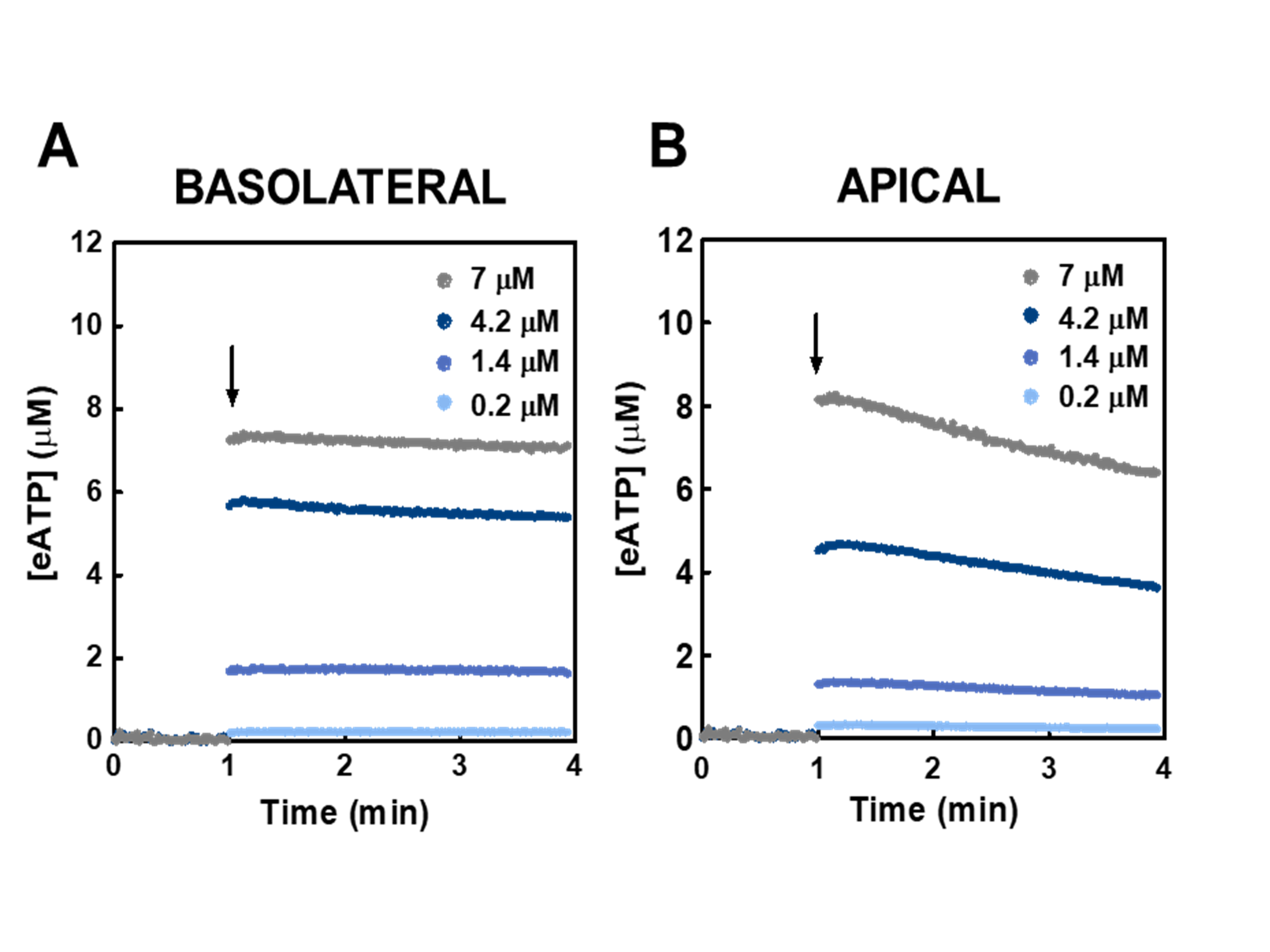

Supplement: S5 Fig — eATP kinetics of cells exposed to [eATP] (0.2–7 μM). Levels of [eATP] were measured by luminometry at the basolateral (A) and apical (B) sides of the polarized Caco-2 monolayers. Data is the mean of 3 independent experiments run in duplicate. The initial velocity of the ecto-ATPase activity was calculated by linear regression to experimental data obtaining the slope and y-intercept of the line. The slope represented the eATP hydrolysis as a function of time, i.e. the ecto-ATPase activity at each [eATP] and in each compartment. (TIF) [file pcbi.1011196.s005.tif]

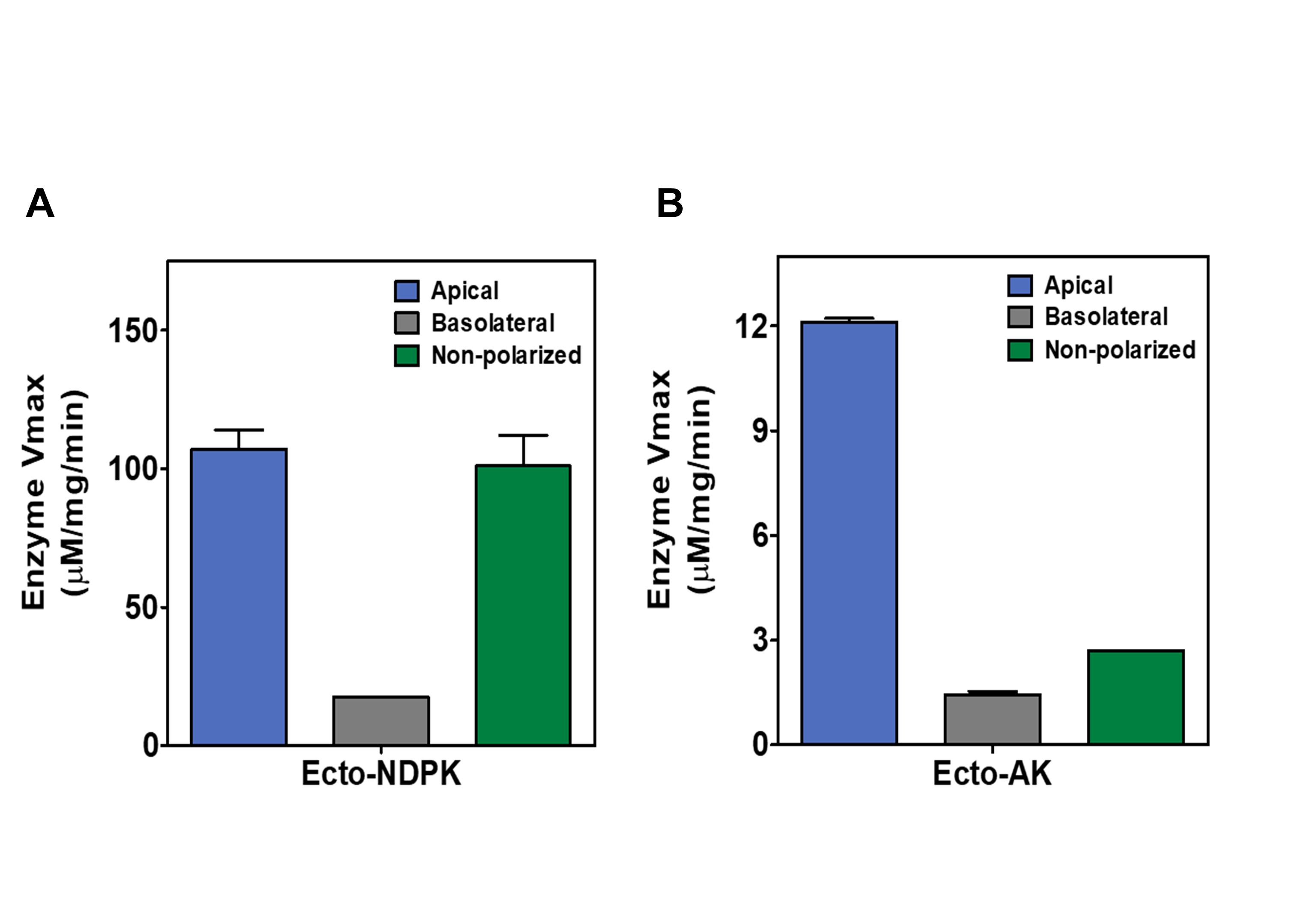

Supplement: S6 Fig — The plot shows the enzymes’ Vmax in the apical and basolateral compartments, and in non-polarized cells. The ecto-NDPK Vmax (A) were obtained from model fitting to experimental data and are the same shown in S2 Table (for the apical and basolateral compartments) and in S1 Table (for the non-polarized cells). The ecto-AK Vmax (B) was calculated from the model parameters using the following formula: FtrAKk-2k1k-2+k1, where the FtrAK was obtained from the model fitting (S2 Table for the apical and basolateral compartments and S1 Table for the non-polarized cells). The k-2 and k1 parameters value can be found in S1 Table. (TIF) [file pcbi.1011196.s006.tif]
